# Improved detection of coronary artery disease by CZT regional coronary blood flow evaluation

**DOI:** 10.3389/fnume.2022.1072729

**Published:** 2022-12-21

**Authors:** R. S. L. Lima, A. Bezerra, M. Andrade, C. Domenico, A. De Lorenzo

**Affiliations:** ^1^ Nuclear Medicine Department, Fonte Imagem, Rio de Janeiro, Brazil; ^2^ Cardiology Department, Universidade Federal do Rio de Janeiro, Rio de Janeiro, Brazil

**Keywords:** myocardial flow reserve, diagnostic accuracy, sestamibi myocardial imaging, cadmium zinc telluride (CdZnTe), coronary artery disease

## Abstract

**Introduction:**

CZT cameras have enabled the noninvasive quantification of myocardial flow reserve (MFR), an important physiologic measure. This study aimed to compare myocardial perfusion SPECT (MPS) with or without MFR evaluation for the detection of obstructive coronary artery disease (CAD).

**Methods:**

48 patients with CAD (>50% obstruction) detected at invasive coronary angiography or CT angiography underwent dipyridamole MPS and MFR evaluation within 30 days. A 1-day protocol (rest-stress) was used to quantify MFR. The acquisition of dynamic rest and stress images was initiated simultaneously to 99mTc sestamibi injection (370 and 1,110 MBq, respectively), both lasting for 11 min, followed by 5-min imaging. Pharmacologic stress with dipyridamole (0.56 mg/kg for 4 min) was performed with the patient positioned in the CZT camera. The images were processed and time-activity curves were generated, calculating global and regional MFR in a semiautomatic software. A global or regional MFR <2 was considered abnormal. MPS perfusion images were classified as normal or abnormal. The images were interpreted by experienced physicians blinded to the results of MFR and coronary angiography/CT.

**Results:**

Mean age of the population was 61 ± 9 years, 54.2% female. Twenty patients (41.7%) had single-vessel CAD, 22 (45.8%) 2-vessel CAD and 6 (12.5%), triple-vessel CAD. Among the 82 vessels with obstruction, 48 had perfusion abnormalities in MPS and 60 had reduced MFR, while among the normal vessels, had 54 normal MPS and 52 had preserved MFR. The sensitivity of MFR (69%) was higher than that of MPS (55.2%), without significant changes in specificity (86 vs. 83.7%).

**Conclusions:**

MFR in the CZT camera is more sensitive for the detection of CAD than perfusion abnormalities in MPS, especially in patients with multivessel CAD.

## Introduction

Cadmium-zinc-telluride (CZT) gammacameras are a relatively recent improvement in the field of Nuclear Cardiology, which have enabled reductions in radiation exposure and scan time (1). The latest opportunity offered by CZT cameras is the possibility of dynamic acquisitions in list mode allowing the measure of both myocardial blood flow (MBF) and myocardial flow reserve (MFR) as in positron emission tomography (PET) imaging (2, 3).

Myocardial blood flow (MBF) is the ratio of the myocardial retention of radiotracers over the integral arterial concentration of tracers, calculated using models which are based on assumptions derived from microsphere modeling and which consider that technetium 99m-labeled tracers are taken up by myocardium according to blood flow (3, 4). MFR is the ratio of MBF during peak hyperemia to MBF at rest. MBF and MFR are powerful clinical tools, which have been increasingly used (5, 6). CZT myocardial perfusion SPECT (MPS) with the measurement of MFR may have higher accuracy for the detection of obstructive coronary artery disease (CAD) than the conventional evaluation of perfusion images (7); nonetheless, evidence is still growing in this field. This study therefore aimed to evaluate the accuracy of conventional perfusion data obtained from CZT MPS compared to CZT MFR for the detection of obstructive CAD in a population of patients referred for functional imaging after definition of the presence of CAD by invasive coronary angiography or computed tomography coronary angiography (CTCA).

## Methods

### Study population

Forty-eight adult patients, referred for MPS by their attending physicians, were studied. All patients were clinically stable and had undergone invasive coronary angiography (29 patients) or CTCA (19 patients) within 30 days before MPS.

Exclusion criteria included contraindications for pharmacological stress with dipyridamole, body mass index ≥40 kg/m^2^, heart failure (New York Heart Association classes III/IV), acute coronary syndrome up to 30 days prior to inclusion in the study, coronary interventions between the determination of coronary anatomy and MPS, extensive coronary calcification precluding optimal CTCA interpretation, and pregnancy. The study was approved by the Institutional Review Board and all participants signed written informed consent.

### Coronary anatomy evaluation

Patients underwent either invasive coronary angiography or CTCA using standard techniques within 30 days before the MPS study. For coronary angiography, two experienced interventional cardiologists classified stenotic lesions visually as percentage of luminal diameter stenosis. A significant obstructive lesion was classified as >50% in a major epicardial artery. Vessels presenting multiple lesions were classified based on the highest degree of stenosis. CTCA studies were performed on a 128-slice scanner (Revolution HD, GE Healthcare, United States) with prospective electrocardiogram (ECG) triggering. Two experienced observers classified stenotic lesions visually as percentage of luminal diameter stenosis. A significant obstructive lesion was classified as >50% in a major epicardial artery. Vessels presenting multiple lesions were classified based on the highest degree of stenosis.

### Study protocol

Patients underwent a 1-day protocol, with rest phase followed by pharmacological stress using dipyridamole. They were instructed to abstain from caffeine, methylxanthines-containing substances and smoking for 24 h before the scan. Additional medications were maintained at the discretion of referring physicians. Scans were performed in a gamma camera with a multi-pinhole collimator and stationary solid-state pixelated detectors made of cadmium-zinc-telluride (Discovery 530, GE Healthcare, Milwaukee, United States) with 99mTc-sestamibi as the radiotracer. To enable positioning of the heart in the camera field of view, a test dose (18.5 MBq) was administered for a pre-scan of 60 s. List mode rest dynamic acquisition was initiated simultaneously to a 30 s manual intravenous injection of 99mTc-sestamibi, in a dose of 370 MBq, followed by a 30 s saline injection, and lasted 11 min with patient positioned in supine position. Routine-gated rest perfusion images were obtained immediately after dynamic acquisition for 5 min. With the patient still positioned inside the camera, dipyridamole intravenous injection was performed at a dose of 0.14 mg/kg/min for 4 min, under electrocardiographic monitoring. At peak stress, a second dose of radiotracer was administered (1,110 MBq) in 30 s, simultaneously to the start of dynamic stress acquisition, also lasting 11 min. Similarly, stress-gated, supine perfusion images were obtained immediately after dynamic stress phase, for 3 min. Aminophylline was injected 11 min after the start of pharmacological stress for all patients. Poststress prone imaging was obtained in all patients.

Static and dynamic data were processed using a dedicated workstation (Xeleris 4.0, GE Healthcare, Haifa, Israel) and commercially available software (Corridor4DM, INVIA Medical Imaging Solutions, Ann Arbor, Michigan, United States). List mode dynamic images were rebinned into 22 frames, consisting of first 18 frames of 10 s (180 s) and four frames of 120 s (480 s). Images were reconstructed using a maximum-likelihood expectation maximization (MLEM) iterative algorithm, with a 3D Butterworth post-filter type, without attenuation or scatter correction. Left ventricular (LV) contours were automatically generated from myocardial images summed from 2 min to the end of the acquisition and a 3D region of interest (ROI) in the middle of the LV was used to sample the blood pool activity. Myocardial uptake was estimated using a generalized net retention model (8, 9). The spillover from the myocardium to the blood pool was set to zero, as it has already been described to be negligible (10). MBF was computed using a flow model for Tc-99 m (10) and MFR was calculated as the ratio of the stress to rest MBF. Subtraction of rest residual activity from the stress dynamic series was performed as previously described (11). Results were reported globally and regionally, as three vascular regions or 17-segment polar map regions. Motion correction was performed to each frame when appropriate. In the current study, the chosen cutoff for MFR was 2.0.

Semiquantitative visual interpretation using a 17-segment model was performed. Segments were scored using a standard five-point system and summed stress (SSS), summed rest (SRS), and summed difference scores (SDS) were obtained. An abnormal study was considered when SSS was >3 (12). The presence of myocardial ischemia (SDS > 1) (12) in each vascular territory was assessed. For the purposes of this study, 2 experts blindly determined the involvement of different coronary territories. Left ventricular ejection fraction (LVEF) was automatically calculated using commercially available software (QGS, Cedars-Sinai Medical Center, Los Angeles, United States).

## Statistical analyses

Continuous variables were expressed as mean ± SD and categorical variables as number (%). The sensitivity and specificity of MPS (defined as the presence of myocardial ischemia in a vascular territory) and of regional MFR for the detection of CAD in each vascular territory were calculated in relation to the presence of obstruction in a given coronary artery.

When analyzing differences between two groups, we applied independent t-test when comparing continuous variables (MBF) and *χ*^2^ test or Fisher's exact test as appropriate when comparing categorial variables (presence of abnormal MPS). Receiver operating characteristic (ROC) areas under the curve were used to evaluate the diagnostic ability of regional MFR by CZT MPS for identifying obstructive CAD. The cut-off on the roc curve was determined at the point that identified the best combination of highest sensitivity and specificity. Analyses were performed using SPSS version 20.0 (IBM Statistics, Armonk, NY, United States).

## Results

**Figure 1:**
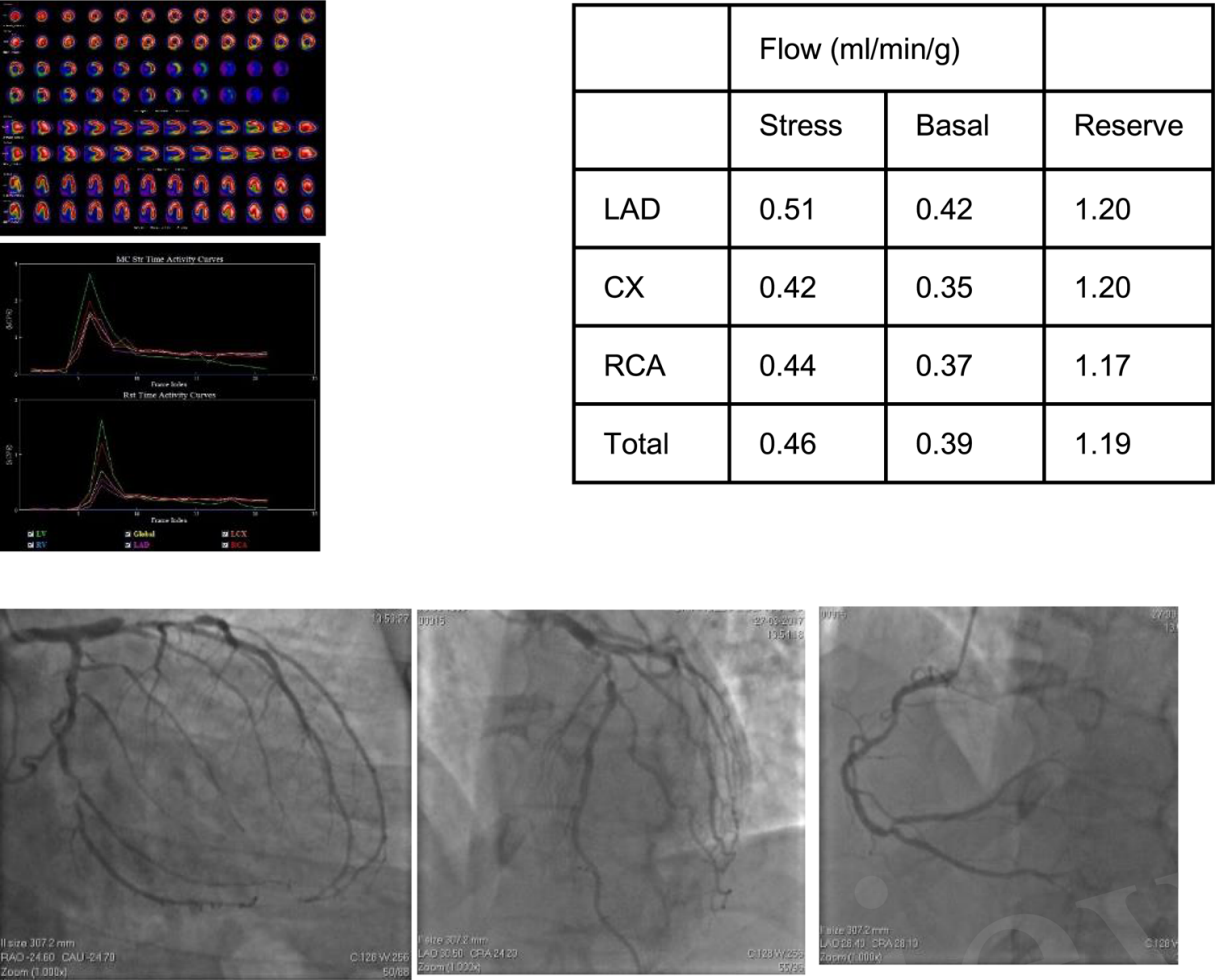
58-year-old male with dyspnea on medium exertion. The clinical history included arterial systemic hypertension, dyslipidemia and a positive family history of CAD. MPI revealed a small fix defect in inferior wall and abnormal MFR in LAD, LCx and RCA territories. Coronary angiography showed a 90% lesion in the proximal LAD, 75% proximal lesion in the second Diagonal, 75% ostial lesions in Marginal 1 and Marginal 3. RCA with a long lesion of 50% in the middle third, in addition to a lesion of 75% in the posterior descending/posterior ventricular bifurcation, compromising the PV in 90%.

Mean age of the population was 61 ± 9 years, and 54.2% were female. Hypertension, dyslipidemia and diabetes were the most frequent risk factors (81.3%, 45.8%, and 43.8%, respectively), and 39.6% had a history of typical angina. The baseline characteristics of the study population are shown in Table 1. Twenty patients (41.7%) had single-vessel CAD, 22 (45.8%) 2-vessel CAD and 6 (12.5%), triple-vessel CAD (Figure 1).

**Table 1:** Baseline characteristics of the study population.

Characteristic	
Age (years)	61 ± 9
Female	26 (54.2)
Body mass index	29.4 ± 4.7
Diabetes	21 (43.8)
Dyslipidemia	22 (45.8)
Hypertension	39 (81.3)
Current smoking	6 (12.5)
Family history of CAD	24 (50)
Typical angina	19 (39.6)
1-vessel CAD	20 (41.7)
2-vessel CAD	22 (45.8)
3-vessel CAD	6 (12.5)

CAD, coronary artery disease.

Numbers are *n* (%) or mean ± SD.

Regarding MPS data, mean SSS was 7.44 ± 4.0 and left ventricular ejection fraction was 50.9 ± 10.1%. The results of MFR evaluation are shown in Table 2.

**Table 2:** Myocardial blood flow analysis.

	Stress	Rest	MFR
LAD	1.93 ± 0.83	0.71 ± 0.36	2.81 ± 0.93
LCX	1.89 ± 0.79	0.65 ± 0.33	3.07 ± 1.16
RCA	1.38 ± 0.54	0.53 ± 0.28	2.76 ± 0.92
Total	1.75 ± 0.7	0.64 ± 0.32	2.86 ± 0.93

Results are ml/min/g.

LAD, left anterior descending coronary artery; LCX, left circumflex coronary artery; MFR, myocardial flow reserve; RCA, right coronary artery.

Among the 82 vessels with obstruction, 48 corresponded to perfusion abnormalities in MPS and 60 had reduced MFR, while among the normal vessels, had 54 normal MPS and 52 had normal MFR (Table 3). The sensitivity of MFR (69%) was higher than that of MPS (55.2%) (*p* < 0.05), without significant changes in specificity (86 vs. 83.7%). Table 4 shows regional baseline and hyperemic MBF and MFR according to the presence of >50% coronary lesions, demonstrating that there are reductions of stress MBF and of MFR, although higher rest MBF in the presence of >50% coronary obstruction compared to <50% stenosis.

**Table 3:** Myocardial flow reserve and MPS results according to the presence of >50% coronary lesions.

	Lesion >50% (*n* = 82)	Lesion <50% (*n* = 62)
MPS- abnormal	48	8
MPS- normal	34	54
MFR < 2.0	60*	10
MFR > 2.0	22	52

MFR, myocardial flow reserve; MPS, myocardial perfusion SPECT.

* *p* < 0.05.

**Table 4:** Regional baseline and hyperemic MBF and MFR according to the presence of >50% coronary lesions.

	Lesion >50% (*n* = 82)	Lesion <50% (*n* = 62)
Stress MBF	1.43 ± 0.68*	1.66 ± 0.7
Rest MBF	0.83 ± 0.42**	0.61 ± 0.30
MFR	1.74 ± 0.42**	2.84 ± 1.06

MBF, myocardial blood flow; MFR, myocardial flow reserve.

* *p* < 0.05.

** *p* < 0.01.

**Figure 2:**
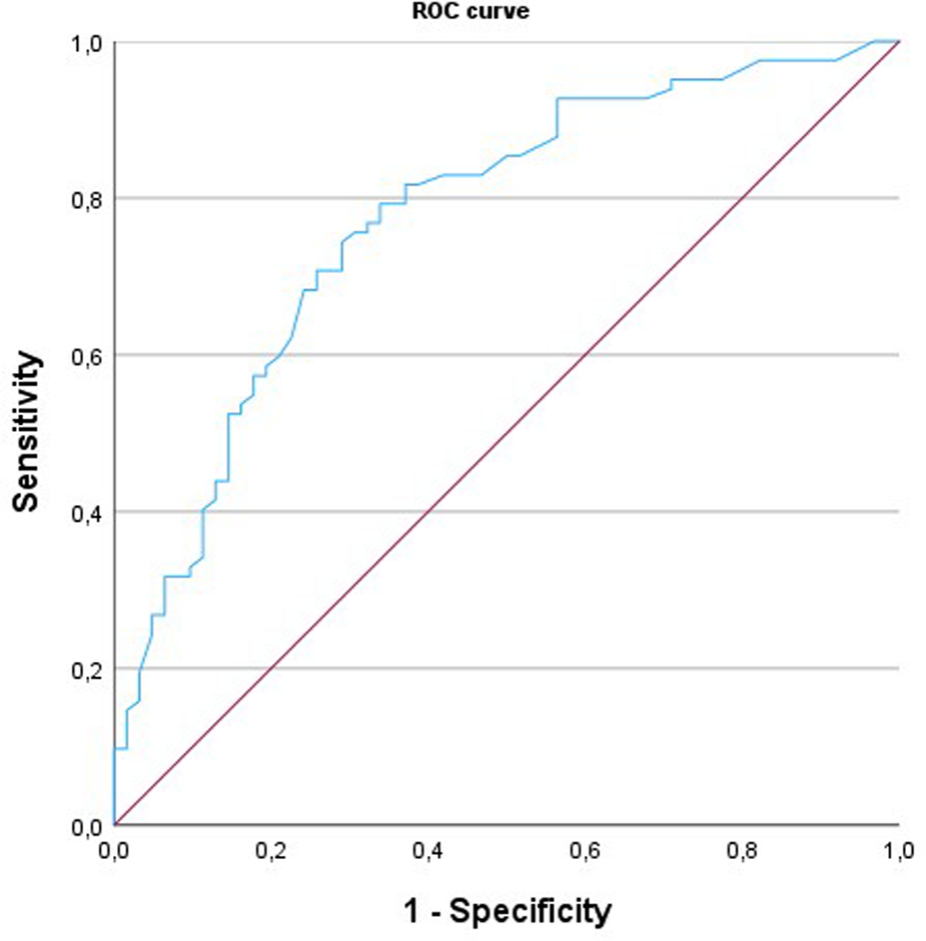
ROC analysis of regional MFR for the identification of >50% coronary artery stenosis.

In the receiver operating characteristic curve analysis (Figure 2), the areas under the curve for the identification of obstructive CAD by MFR were 0.772 (95% CI: 0.70–0.85). A regional MFR of 2.0 provided the best trade-off between sensitivity and specificity for identifying obstructive CAD.

## Discussion

The noninvasive assessment of myocardial blood flow has been the subject of a growing research effort, initially with PET, followed by SPECT, the latter enabled by CZT cameras (13–15). Images obtained from dynamic CZT SPECT have high quality, temporal and spatial resolutions (1). Articles regarding MBF and MFR measurement using this cardiac-dedicated SPECT have demonstrated encouraging results, with good reproducibility and accuracy (4, 6, 16–18). Nonetheless, continuing experience with this technology is needed, as MPS is overall more widely available than PET.

In some previous studies to evaluate MBF, doses of radiotracer were used according to the patient's weight. In the present study, we used a fixed dose because in a previous study to evaluate the protocol this form was used, but this aspect of using the lowest possible exposure dose is fundamental. Acampa et al. tested a low-dose dynamic myocardial perfusion imaging by CZT-SPECT with similar results (19).

In this study, in 82 vessels with coronary obstruction, 48 had perfusion abnormalities in MPS and 60 had reduced MFR, while among the normal vessels, had 54 normal MPS and 52 had preserved MFR. When >50% coronary stenosis was present, stress MBF and MFR were reduced when compared to <50% lesions; rest MBF was higher in the former, possibly due to the use of medications, which were not evaluated in this study. Also, hemodynamic data were not available, limiting the interpretation of this finding, as hemodynamic parameters may affect rest MBF.

The sensitivity of MFR for the detection of obstructive CAD (69%) was higher than that of MPS (55.2%), without significant changes in specificity (86 vs. 83.7%, respectively). This is of great clinical value, as MPS is a common gatekeeper for coronary angiography (20). In the current study, the chosen cutoff for MFR was 2.0. A previous study from our group (21) showed that, using ROC curve analysis, a global MFR cutpoint of 2.08 provided a sensitivity of 66.7% and a specificity of 84.6% for the diagnosis of high-risk CAD. A regional MFR cutoff point of 2.2 provided a sensitivity of 63.2% and specificity of 74.1% for the identification of an obstructive lesion in the corresponding artery. Prior evidence also showed improved detection of multivessel CAD with the 2.0 MFR cutoff (22). Additionally, the definition of obstructive CAD was 50% obstruction (contrary to the prior 70% definition). Therefore, the higher sensitivity of MFR found in this study (69% compared to 63.2%) with a lower cutoff of obstruction (50% vs. 70%) shows an even better performance of MFR in this study. The increased sensitivity is very important to overcome one of the greatest pitfalls of MPS, which is the reduced sensitivity to detect multivessel CAD (23).

A recent study by Le et al. (24) demonstrated, in patients with single-vessel CAD, that MPS coronary flow reserve (CFR) was useful for the detection of functionally significant stenosis defined by fractional flow reserve (FFR). In our study, most patients had multivessel CAD disease, turning it a population that could have balanced ischemia, which is known to hinder the identification of individual territories with perfusion abnormalities in MPS; thus, MFR enabled an increase of sensitivity for detection of abnormal regional perfusion compared to MPS.

Finally, it is worth noting that vascular function is influenced by other factors than major coronary artery anatomy, such as microvascular disease (25), which would be of concern in this study due to the high percentage of diabetic patients; however, considering that in the current study all patients had documented coronary anatomy, the presence of flow abnormalities can be more easily attributed to macrovascular disease than to microcirculatory dysfunction.

## Limitations

The main limitation of this study is that the sensitivity and specificity of MPS may have been influenced by the fact that all participants had obstructive CAD. As MFR may be reduced in the absence of obstructive CAD, other studies might have experienced an overall reduction of specificity, which did not happen in this work. Additionally, baseline differences of MBF might be attributed to the use of medications or hemodynamic parameters, which were not evaluated in this study. Finally, this is a single-center study with a relatively small number of participants. Further investigations, especially if multicenter, may help underscore our findings.

## Conclusions

In this population with documented CAD, the evaluation of MFR in the CZT camera was more sensitive for the detection of CAD than perfusion abnormalities in MPS, especially in patients with multivessel CAD.

## Data Availability

The original contributions presented in the study are included in the article/Supplementary Material, further inquiries can be directed to the corresponding author.
